# Supplementary figures and images for: Deep mutational scanning reveals pharmacologically relevant insights into TYK2 signaling and disease
Source: eLife. 2026 Jun 10;15:RP110149. doi: 10.7554/eLife.110149 (PMC13252953; doi:10.7554/eLife.110149)

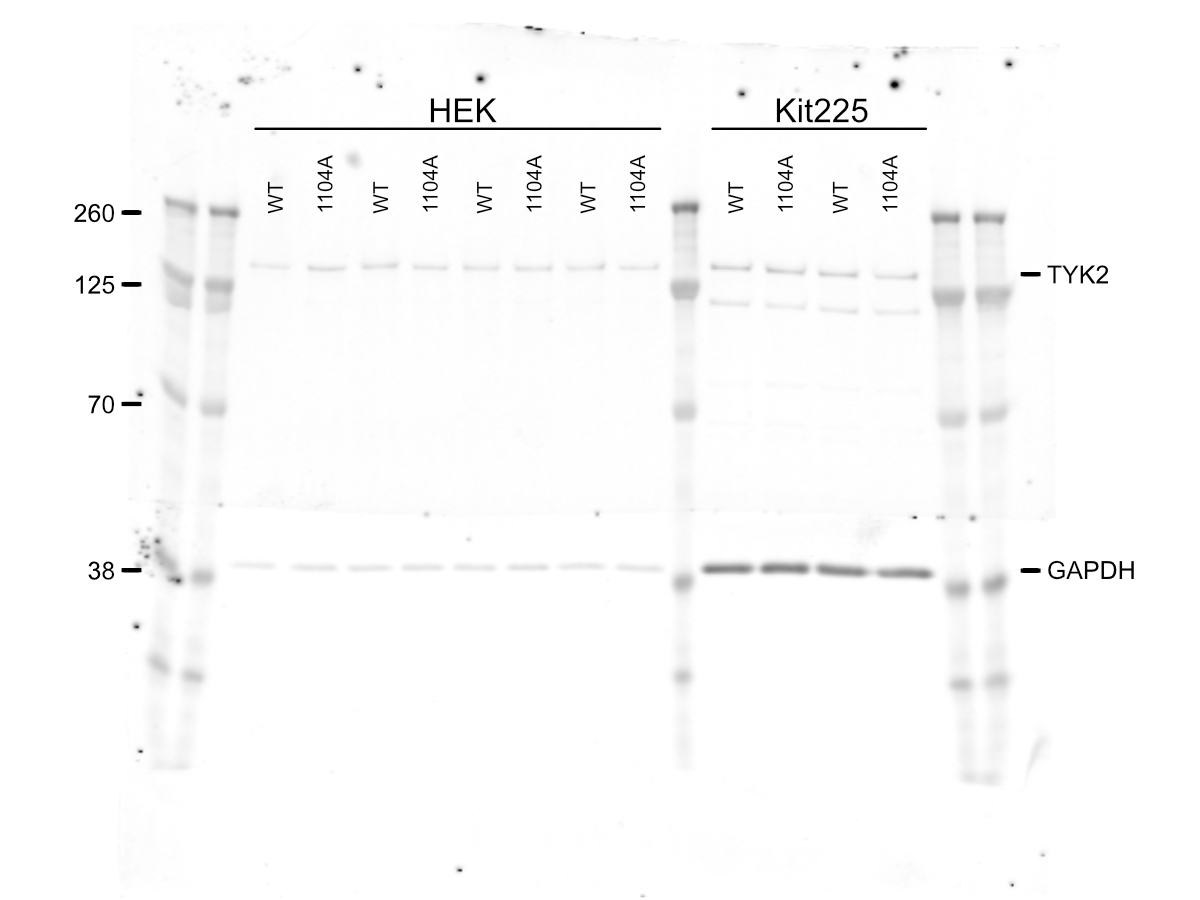

Supplement: Figure 4—figure supplement 1—source data 1. [file elife-110149-fig4-figsupp1-data1.zip › SupplementaryFigure6_SourceData_1.jpg]

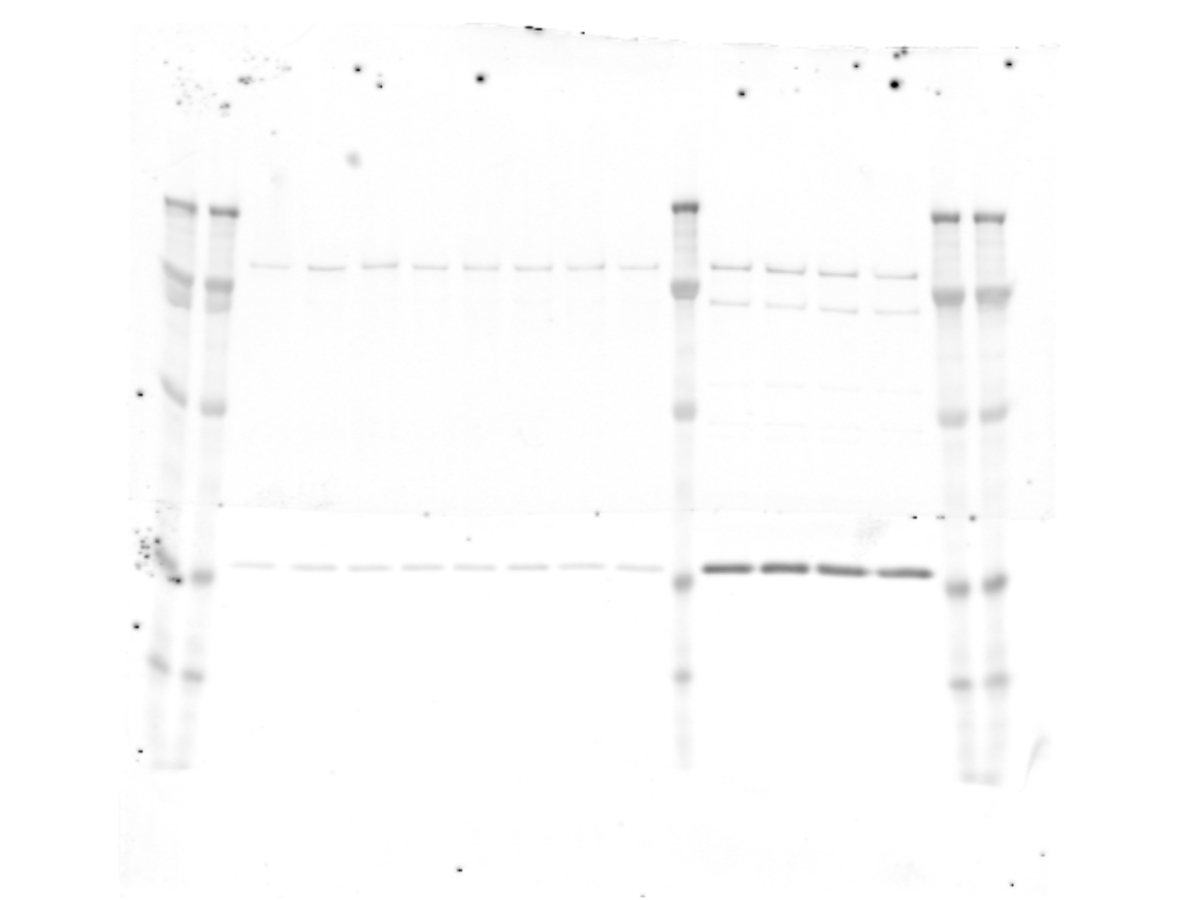

Supplement: Figure 4—figure supplement 1—source data 2. [file elife-110149-fig4-figsupp1-data2.zip › SupplementaryFigure6_SourceData_2.jpg]

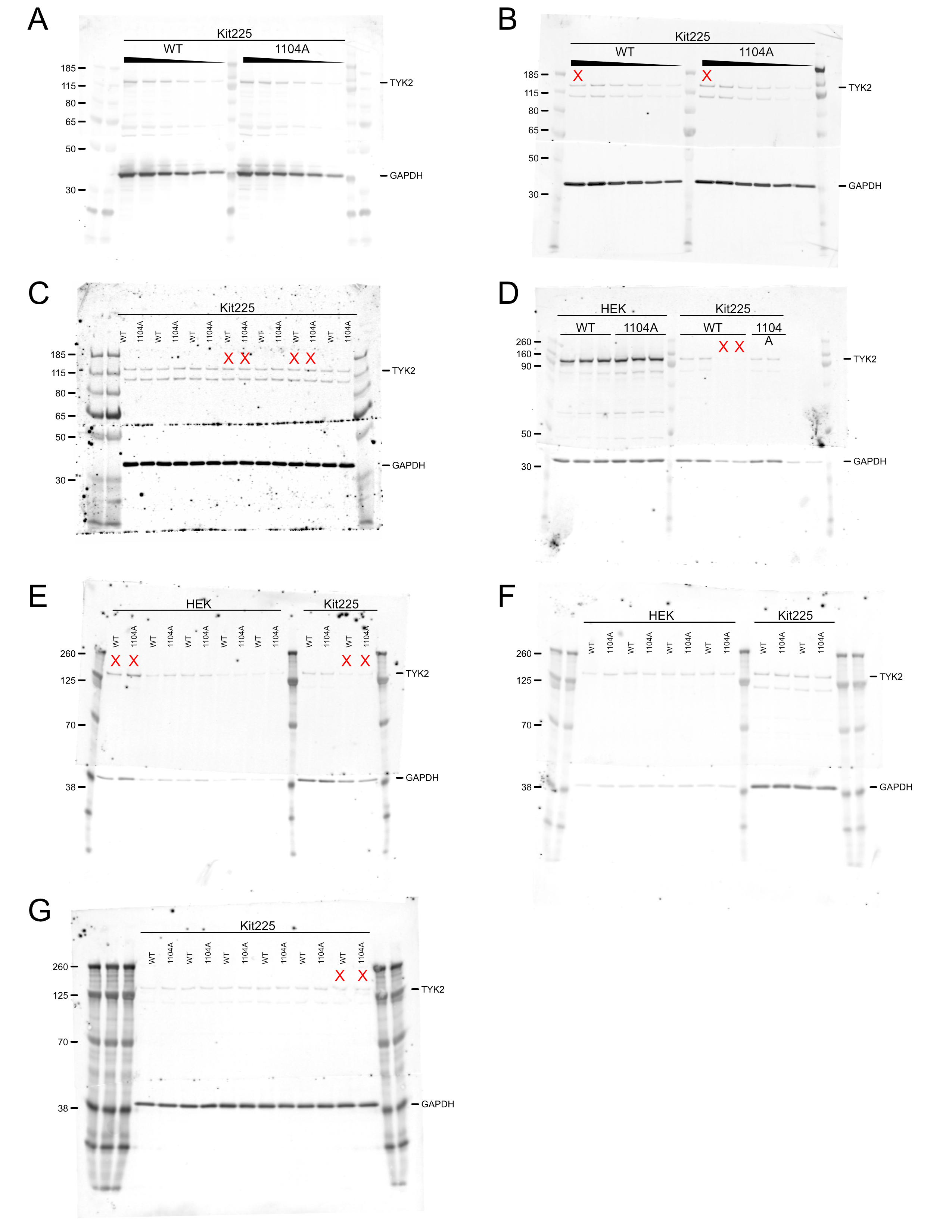

Supplement: Figure 4—figure supplement 3—source data 1. [file elife-110149-fig4-figsupp3-data1.zip › SupplementaryFigure8_SourceData_1.jpg]

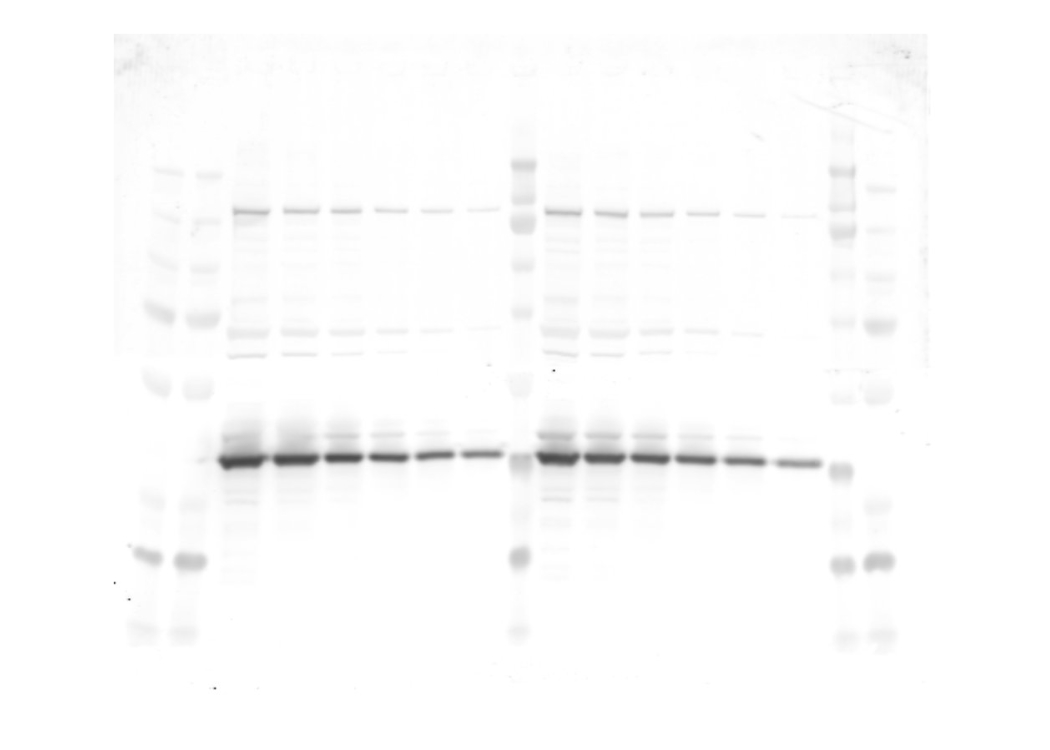

Supplement: Figure 4—figure supplement 3—source data 2. [file elife-110149-fig4-figsupp3-data2.zip › SupplementaryFigure_8A.jpg]

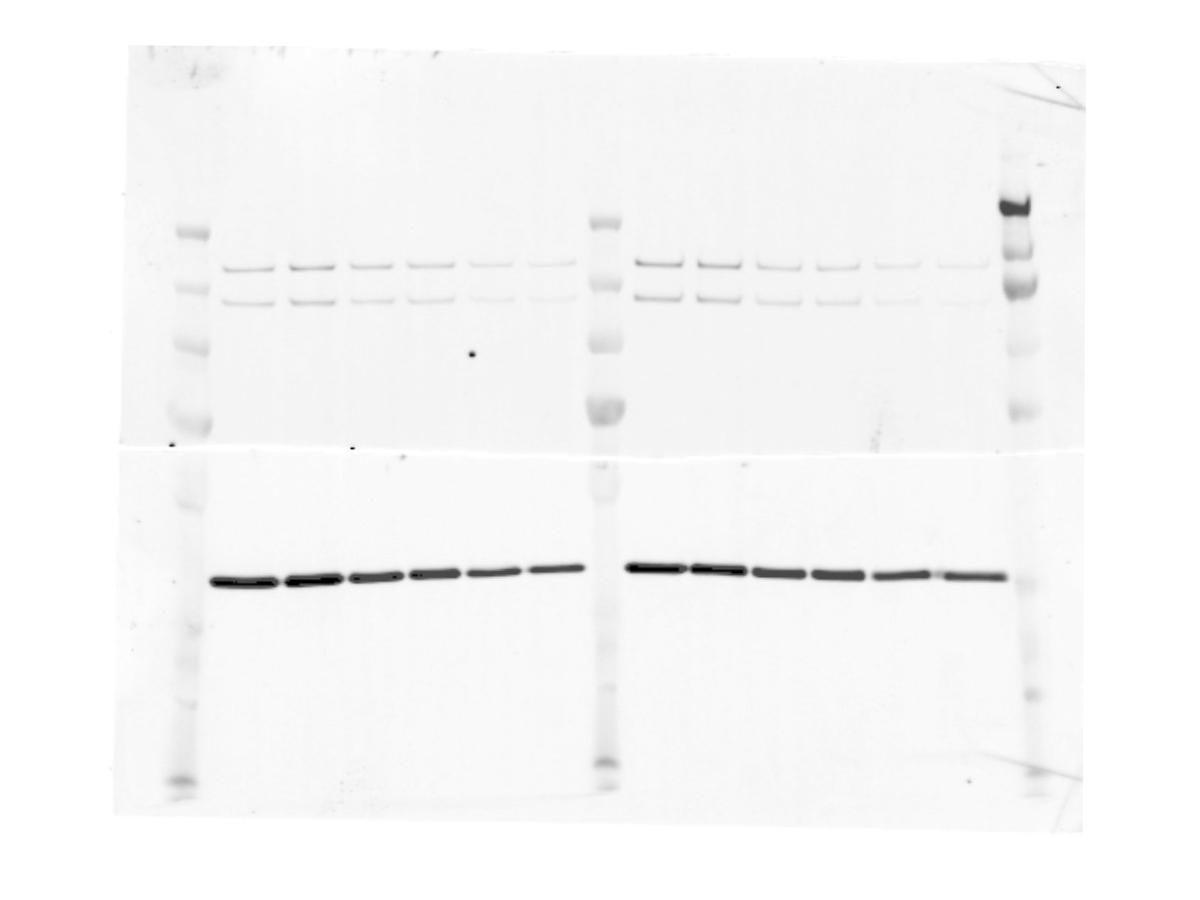

Supplement: Figure 4—figure supplement 3—source data 2. [file elife-110149-fig4-figsupp3-data2.zip › SupplementaryFigure_8B.jpg]

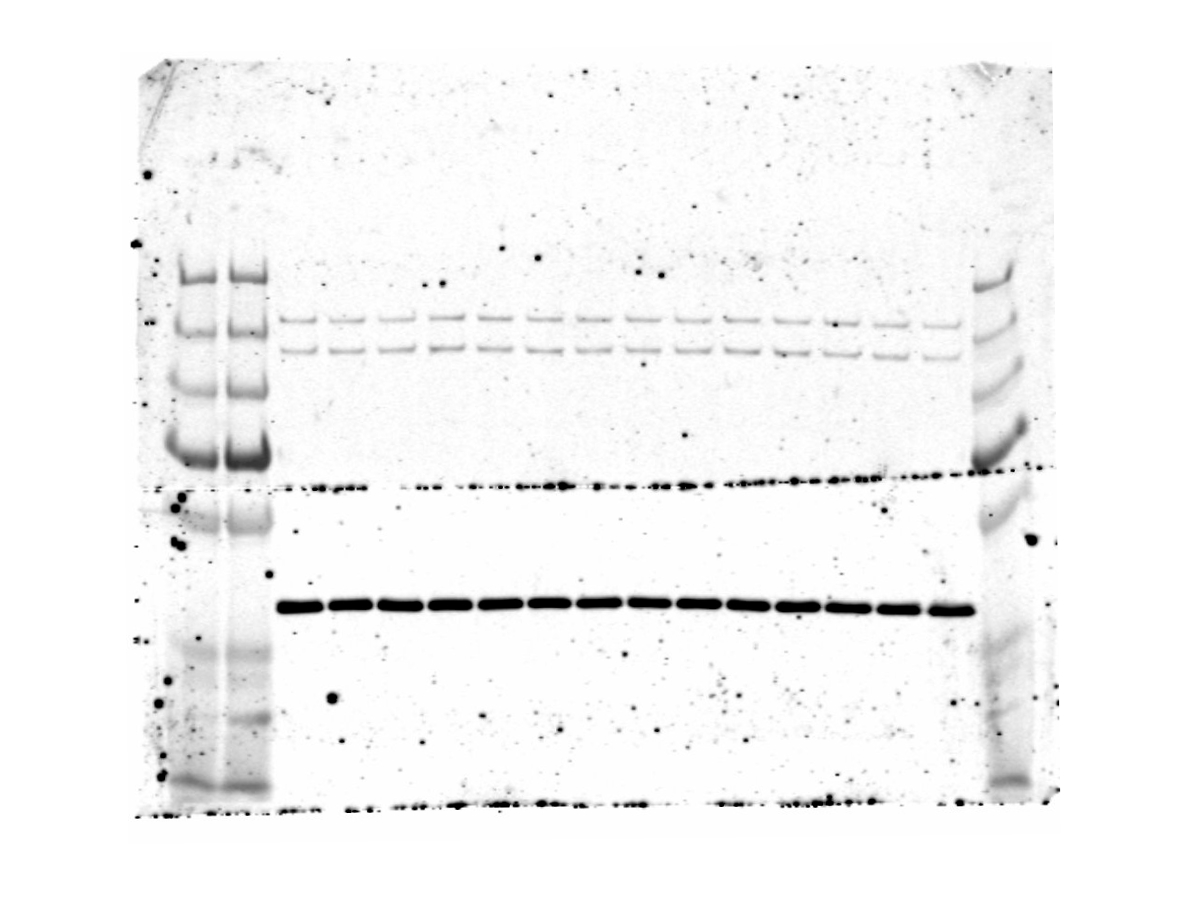

Supplement: Figure 4—figure supplement 3—source data 2. [file elife-110149-fig4-figsupp3-data2.zip › SupplementaryFigure_8C.jpg]

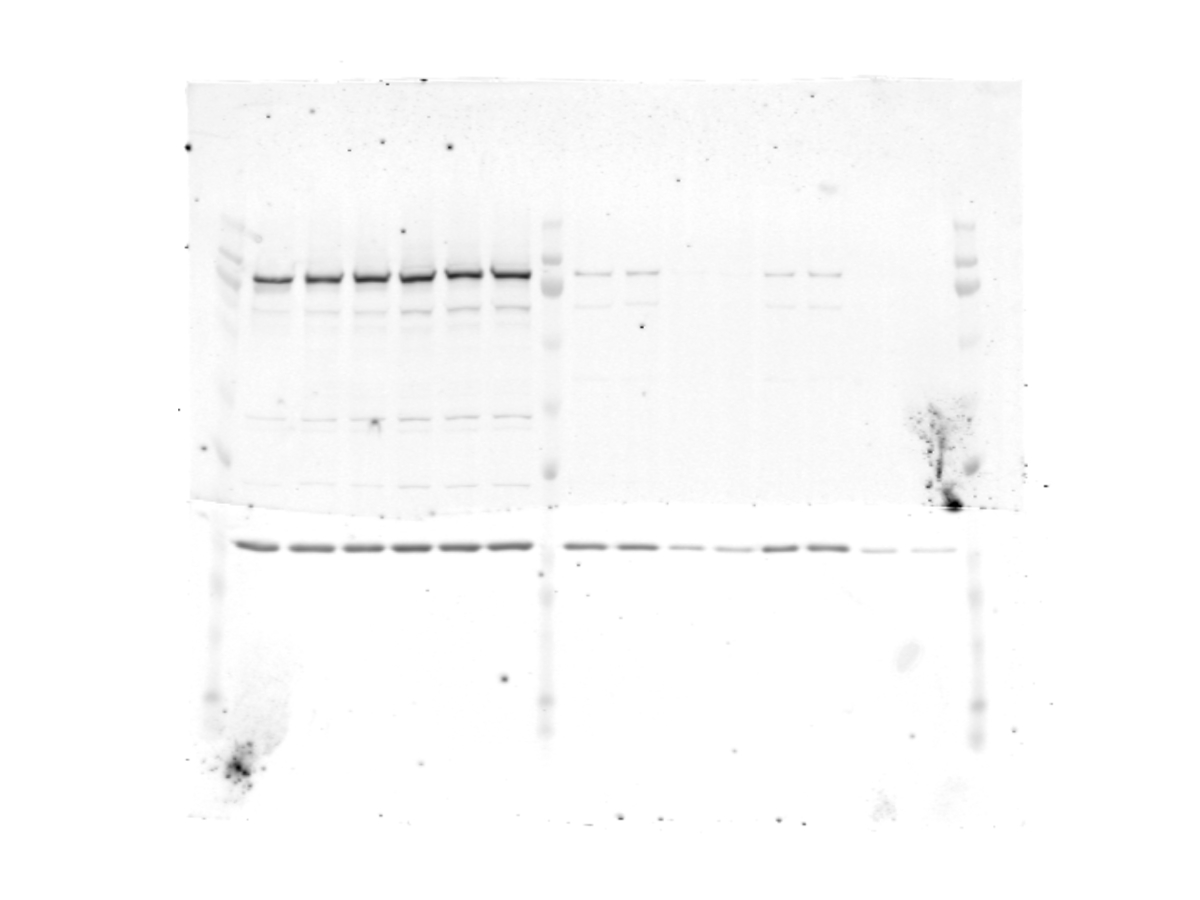

Supplement: Figure 4—figure supplement 3—source data 2. [file elife-110149-fig4-figsupp3-data2.zip › SupplementaryFigure_8D.jpg]

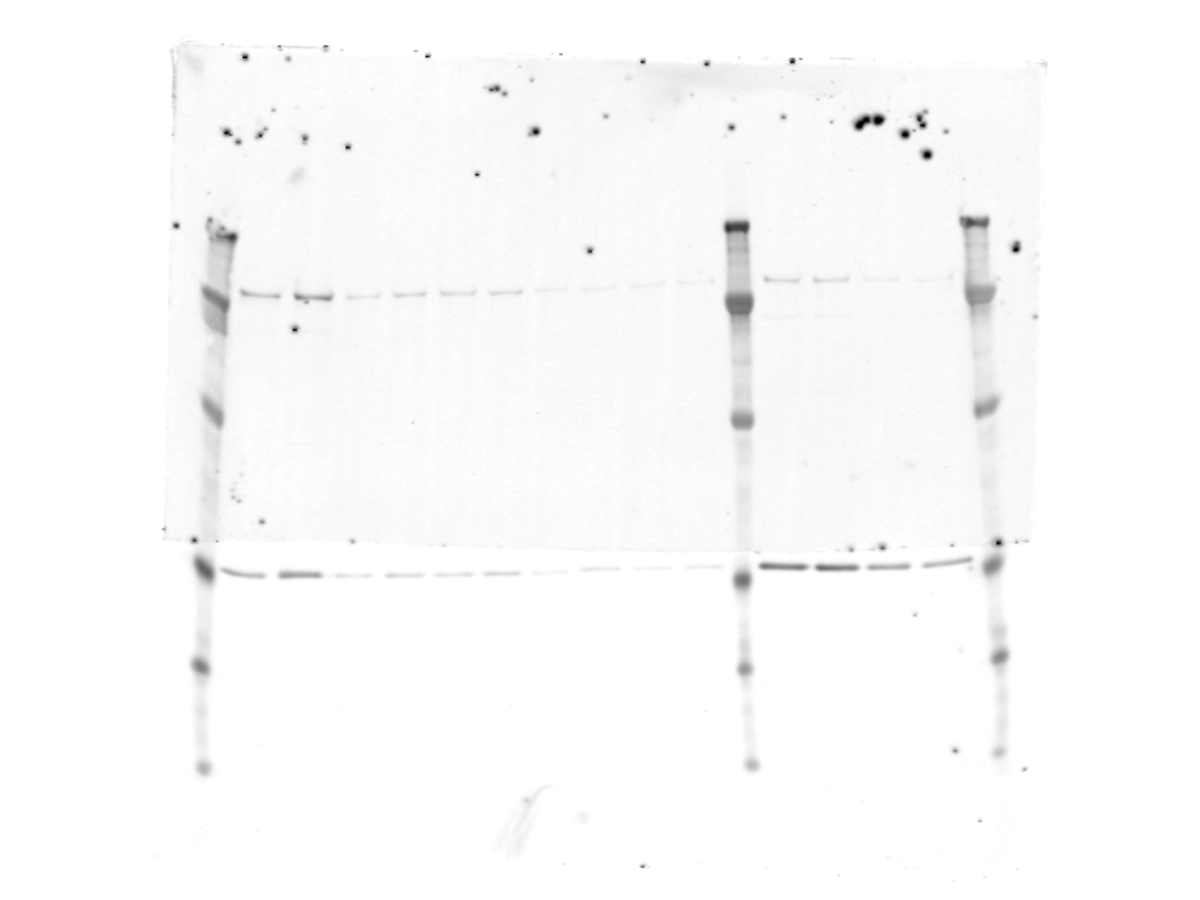

Supplement: Figure 4—figure supplement 3—source data 2. [file elife-110149-fig4-figsupp3-data2.zip › SupplementaryFigure_8E.jpg]

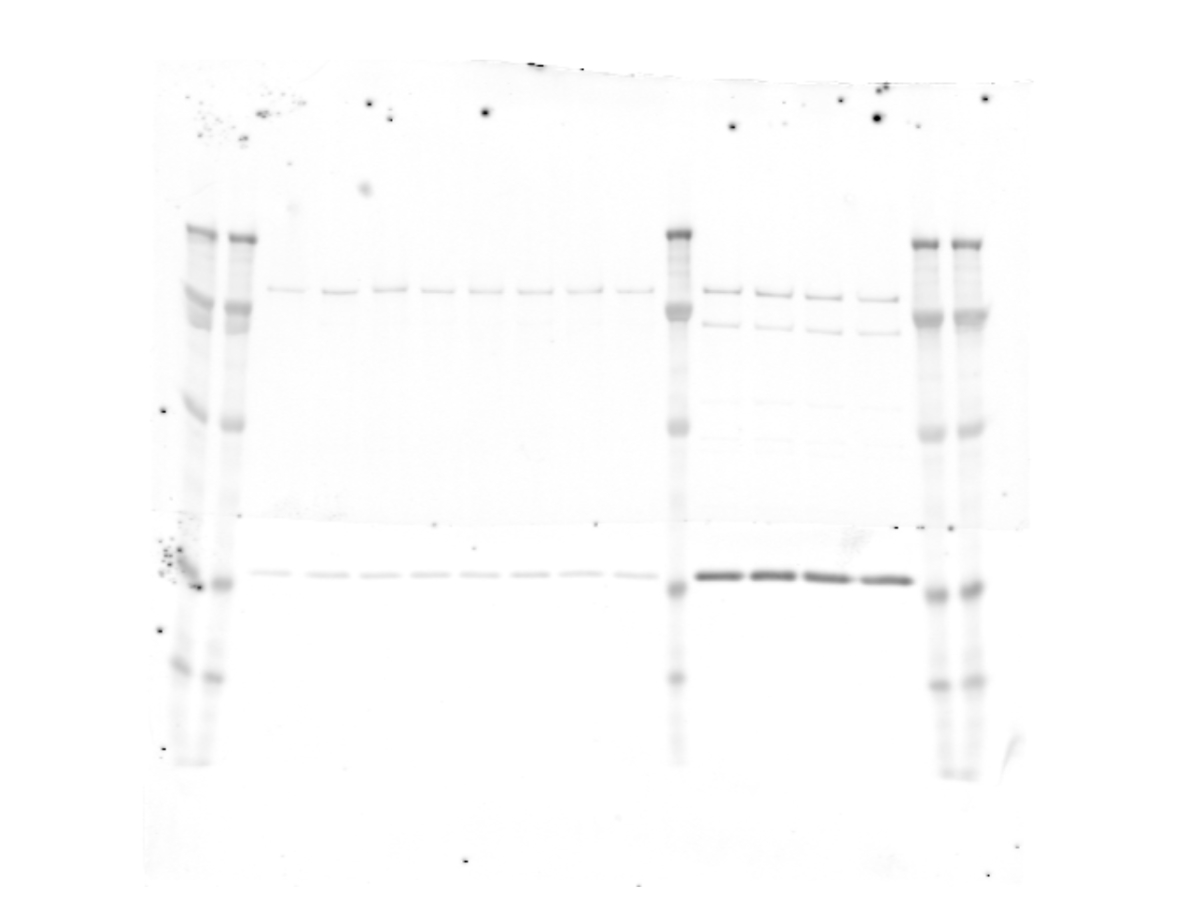

Supplement: Figure 4—figure supplement 3—source data 2. [file elife-110149-fig4-figsupp3-data2.zip › SupplementaryFigure_8F.jpg]

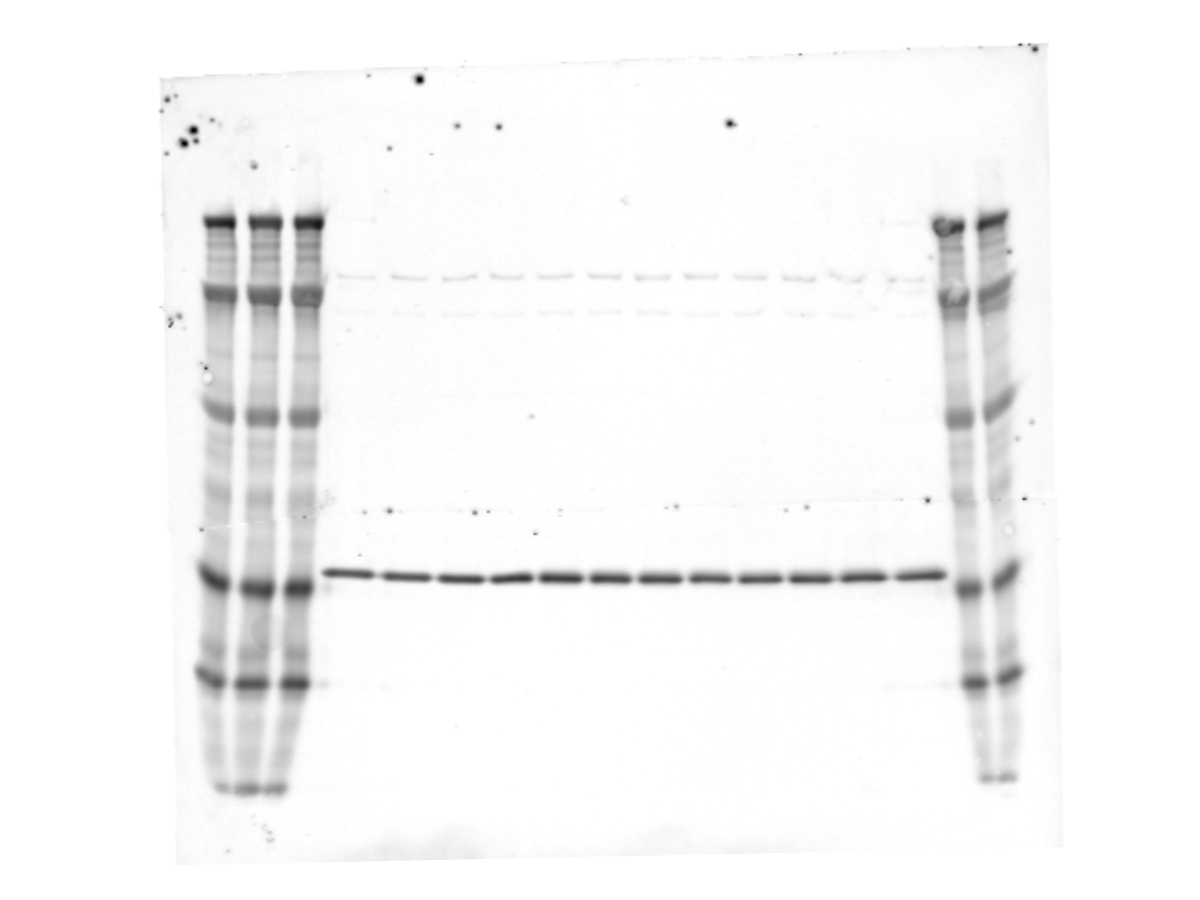

Supplement: Figure 4—figure supplement 3—source data 2. [file elife-110149-fig4-figsupp3-data2.zip › SupplementaryFigure_8G.jpg]
