# Supplementary material for: Deep mutational scanning reveals pharmacologically relevant insights into TYK2 signaling and disease
Source: eLife. 2026 Jun 10;15:RP110149. doi: 10.7554/eLife.110149 (PMC13252953; doi:10.7554/eLife.110149)
Supplement: Peer review history

# Editor report: eLife Assessment

**DOI:** 10.7554/eLife.110149.2.sa0

**Authors:** Markus A Seeliger

**Affiliations:** https://ror.org/05qghxh33 Stony Brook University United States

**Keywords:** Compelling, Important

This **important** paper presents a rigorous and comprehensive deep mutational analysis of the kinase TYK2, revealing how single amino acid substitutions influence protein abundance, signaling activity, and responses to pharmacological inhibitors. By combining high‑quality experimental design with dose‑response signaling assays and multiple inhibitor conditions, the authors generate a robust dataset that identifies variants across all domains of TYK2, including clusters at functionally critical sites and protein-protein interfaces. The study highlights mutations that drive drug resistance or potentiation and shows that reduced TYK2 abundance aligns with protective autoimmune‑associated variants, underscoring the therapeutic relevance of modulating TYK2 stability. Overall, the work provides **compelling** insights with clear implications for biochemistry, immunology, clinical genetics, and drug discovery.

# Referee report: Reviewer #1 (Public review)

**DOI:** 10.7554/eLife.110149.2.sa1

Summary:

In this compelling study, Howard et al. use deep mutational scanning to probe essentially all possible single amino acid substitutions in the TYK2 tyrosine kinase, and identify those that modulate signaling function and protein abundance. The methodological approach is elegant and thorough, and the results identify numerous examples of amino acid substitutions that have been previously reported to modulate TYK2 function, validating the approach.

Substitutions that are LOF with respect to IFN-a signaling but not protein abundance are particularly interesting and are widely dispersed across the protein. They include known functionally critical sites such as the active site and activation loop of the kinase domain, as well as the allosteric site within the regulatory pseudokinase domain, but also hundreds of other additional sites. The approach is then used to study the effects of substitutions on kinase inhibition using several JAK family inhibitors that target the pseudokinase domain. By assessing variant effects at both high and low drug concentrations, they are able to identify variants that mediate resistance or conversely potentiate inhibition, respectively. These map to distinct sites on the pseudokinase domain. Finally, the authors show that several TYK2 variants, most notably the P1104A substitution, previously shown to protect against autoimmune disease, correspond to substitutions that reduce protein abundance in their screen. Combining their DMS data with autoimmune phenotype and TYK2 genotype data uncovered a general dose relationship between autoimmunity and TYK2 abundance, and the authors propose that this might justify targeting TYK2 protein levels with degraders.

Strengths:

This is a nicely executed, well-written study with good figures and a clear presentation.

Weaknesses:

The only substantial critique I have is that while the paper makes a compelling case for the validity and power of the approach, the authors could perhaps go further in their interpretation of their data, particularly with regards to identifying functionally important sites and connecting them to putative allosteric sites and functionally relevant protein-protein interfaces in the context of what is known about JAK family kinase structure and function. An attempt is made to interpret the data in light of a composite structural model of full-length TYK2 engaged with the IFNAR1 receptor (Figure 2C), but much more could be said about this. Below, I list several examples where additional insight might be gleaned.

(1) The discussion of gain-of-function variants is limited. Given that tight regulation is a general theme of kinase signaling and gain-of-function mutations are a common disease mechanism, these mutations could be particularly interesting. Could the authors comment on patterns of gain versus loss? Are there gain-of-function signaling variants that work in a IFN-a dose dependent versus independent manner?

(2) The discussion of the signaling-specific variants (LOF in signaling but not abundance) is interesting but could be expanded. Can the authors comment on which regions of the pseudokinase/kinase interface, for instance, are affected, since this allosteric communication is a critical and unique aspect of JAK family protein function? Can something be said about what the 6 activation loop substitutions are doing?

(3) The cytokine signaling screen was performed at several different levels of IFN-α cytokine stimulation. The authors state that these data were used to identify quantitative variant effects (p7), but the cytokine dose response data are not widely discussed in the manuscript. Is it not possible that valuable information about the strength of substitution effects could be gleaned from this? One might expect that simple loss of function mutants that, e.g. completely destroy catalytic activity, will be LOF at all levels of stimulation, whereas mutations that have more nuanced "tuning" or allosteric effects on signaling might display LOF at low cytokine stimulation levels but be restored at high stimulation levels. Such information could be of potential functional importance and interest. Could the authors comment on this?

(4) In general, the variant data could be interpreted more specifically in light of the available detailed structural information about TYK2 and JAK kinases generally. For instance, could the resistance versus potentiation variants be interpreted in this context to hypothesize what they might be doing?

# Referee report: Reviewer #2 (Public review)

**DOI:** 10.7554/eLife.110149.2.sa2

Howard et al. describe a set of deep mutational scanning (DMS) experiments applied to TYK2, which is a drug target implicated in autoimmune disease. By assaying protein abundance (stability) effects as well as immune signaling, the authors are able to disentangle variant effects that may be directly involved in protein activity (and therefore potentially druggable) from variant effects that are due to loss of protein or general structural instability. By performing these assays under multiple conditions, including the presence of various concentrations of small molecules, they develop a clear picture of which sites in TYK2 may be most relevant for intervention or targeting. Overall, the work represents a very compelling example of DMS for understanding protein biology and candidate drug mechanisms.

The work is very thorough, with multiple DMS assays described and compared/contrasted. This greatly enhances the impact and interpretability of any individual assay performed.

The authors have made improvements to the state of the art in terms of wet-lab assay design as well as the analysis of FACS-based deep mutational scans.

The potential mechanism of loss of protein abundance in TYK2 being protective for autoimmune disease is clear, but the estimates of the effect size in more physiologically relevant settings vary quite a bit and might be quite small. Are there examples that could be cited of other similar disease mechanisms where a 10% loss in abundance is associated with a clinical phenotype?

# Referee report: Reviewer #3 (Public review)

**DOI:** 10.7554/eLife.110149.2.sa3

Summary:

In the paper "Deep mutational scanning reveals pharmacologically relevant insights into TYK2 signaling and disease", the authors perform a comprehensive deep mutational scan of the kinase TYK2, a protein of pharmacological interest due to its central role in multiple immune-related phenotypes. The study assesses two key functional phenotypes: protein abundance and IFN-α-dependent signaling. The signaling assays were conducted across a dose-response range under various inhibitor conditions, allowing for an in-depth characterization of TYK2 activity and regulation. Both the experimental design and data analysis were executed with rigor and transparency, yielding a dataset that appears highly reliable. The authors provide strong evidence and a scientifically grounded interpretation of their results.

The paper presents the results of a deep mutational scan based on two assays: an IFN-α-stimulated signaling assay and a protein abundance assay. These measurements are further supported by variant classifications from AlphaMissense and ClinVar, providing a framework for functional interpretation. Building on these data, the authors propose four potential pharmacological applications of their screening system at the end of the first results section.

First, they demonstrate that the combined analysis of abundance and IFN-α signaling identifies potential allosteric sites, focusing on variants with normal protein stability but reduced signaling activity. Through this approach, they detect two previously uncharacterized allosteric regions (Results Section 2).

Second, they explore how the screen can be used to predict variant-specific drug responses or resistance mechanisms (Results Section 3). This is achieved through assays involving two different inhibitors, which reveal both resistance- and potentiation-associated variants.

Third, they assess the relative functional consequences of ligand and inhibitor dosing by performing IFN-α and inhibitor dose-response experiments (1, 10, and 100 U/mL IFN-α; IC99 and IC75 inhibitor concentrations; Results Section 3).

Finally, the authors investigate how specific human variants, such as P1104A and I684S, may inform therapeutic modality selection (Results Section 4). Although these variants exhibit no detectable effect on IFN-α signaling within this experimental system, they substantially impact protein abundance. By integrating data from the UK Biobank, the authors further demonstrate that protective effects against autoimmune disease are associated with altered protein abundance rather than differences in IFN-α signaling, highlighting the distinct mechanistic basis of TYK2's clinical relevance.

Strengths:

Overall, we found this paper rigorous, well-written, and easy to follow. As such, we think this is an exceptional example of a deep mutational scanning manuscript, and this dataset will be invaluable to the field. We particularly appreciate that the authors could explore sensitivity to inhibitor concentration across multiple doses of the inhibitor.

Weaknesses:

Despite the authors' rigorous experimentation and thoughtful interpretation, the study leaves several important mechanistic questions unresolved, as is common in any study. While the data provide clear functional patterns, the underlying biophysical and biochemical explanations remain insufficiently explored. For instance, in point 1, the identification of two novel allosteric sites is intriguing, yet the paper does not elaborate on the structural basis or mechanistic rationale for their regulatory effects. In point 2, resistance and potentiation variants are described for two distinct inhibitors, but it remains unclear why certain variants respond specifically to one compound and not the other. In point 3, higher inhibitor concentrations appear to diminish allosteric interactions, though the reasons why some sites are affected while others are not are left unexplained. Finally, in point 4, the observation that protein abundance, but not IFN-α signaling, correlates with autoimmune protection is compelling but mechanistically ambiguous. These gaps do not detract from the technical excellence of the work; rather, they highlight opportunities for future studies to clarify the molecular and pharmacological mechanisms underlying TYK2 regulation and to deepen the translational insights drawn from this comprehensive mutational scan. We hope that the authors could provide more direction and mechanistic context in the discussion section to guide readers toward these next steps.

# Author comment: Author response

**DOI:** 10.7554/eLife.110149.2.sa4

**Authors:** Conor J Howard, Nathan S Abell, Robert R Warneford-Thomson, Eden Mahdavi, Alan L Su, Carmen Resnick, Nabil Mohammed, Erin M Thompson, Emily R Holzinger, Katrina Catalano, Abhay Hukku, Gabriel A Mintier, Morgan MacKenzie, Bryan L Jiang, Dora Barbosa Rabago, Angela Chan, Carolindah Ntimi, Kaitlyn N Weiler, Stephen C Wilson, Joseph Maranville, Payal R Sheth, Robert M Plenge, Sriram Kosuri, Diane E Dickel

**Affiliations:** Octant, Inc Emeryville United States; Octant, Inc Emeryville United States; Octant, Inc Emeryville United States; Octant, Inc Emeryville United States; Octant, Inc Emeryville United States; Octant, Inc Emeryville United States; Octant, Inc Emeryville United States; Octant, Inc Emeryville United States; Bristol Myers Squibb New York United States; Bristol Myers Squibb New York United States; Bristol Myers Squibb New York United States; Bristol Myers Squibb New York United States; Octant, Inc Emeryville United States; Octant, Inc Emeryville United States; Octant, Inc Emeryville United States; Bristol Myers Squibb New York United States; Bristol Myers Squibb New York United States; Bristol Myers Squibb New York United States; Bristol Myers Squibb New York United States; Bristol Myers Squibb New York United States; Bristol Myers Squibb New York United States; Bristol Myers Squibb New York United States; Octant, Inc Emeryville United States; Octant, Inc Emeryville United States

We thank the reviewers for their excellent and thoughtful comments and suggestions, along with their strong support of the work. We agree with the general feedback that there is opportunity for further mechanistic dissection of the data from a variety of interesting angles. This was a fascinating project to work on because of all of the possible directions, and we attempted to highlight a diversity of compelling findings. We wish we had time to devote to answering more of the open mechanistic questions, but, given competing priorities, we are unfortunately unable to do them justice at this time. At the suggestion of a reviewer, we have made results available through MaveDB (accession numbers urn:mavedb:00001270-a and urn:mavedb:00001271-a) as a way to empower others to explore more.
